# The alterations in nerve growth factor concentration in plasma and synovial fluid before and after total knee arthroplasty

**DOI:** 10.1038/s41598-024-59685-1

**Published:** 2024-04-18

**Authors:** Tomohiro Onodera, Koji Iwasaki, Masatake Matsuoka, Yasuhide Morioka, Shinji Matsubara, Eiji Kondo, Norimasa Iwasaki

**Affiliations:** 1 Department of Orthopaedic Surgery, Faculty of Medicine and Graduate School of Medicine, Hokkaido University, N15W7, Sapporo, Japan; 2 Department of Functional Reconstruction for the Knee Joint, Hokkaido University, N15W7, Sapporo, Japan; 3 Laboratory for Drug Discovery and Disease Research, Shionogi & Co. Ltd, Osaka, Japan; 4 Centre for Sports Medicine, Hokkaido University, N14W5, Sapporo, Japan

**Keywords:** Total knee arthroplasty, Osteoarthritis, Postoperative pain, Nerve growth factor, Biomarkers, Diseases, Medical research, Rheumatology

## Abstract

Total knee arthroplasty (TKA) is an effective procedure for pain relief; however, the emergence of postsurgical pain remains a concern. In this study, we investigated the production of nerve growth factor (NGF) and mediators that affect NGF production and their function in the synovial fluid and plasma after TKA. This study included 19 patients (20 knees) who had rheumatoid arthritis (RA), systemic lupus erythematosus (SLE), and knee osteoarthritis (OA) who underwent TKA, categorized into OA and non-OA groups. The levels of NGF, inflammatory cytokines, and lipid mediators were analyzed before and after surgery. The intraoperative synovial fluid NGF concentration was more than seven times higher in the non-OA group than in the OA group. The intra-articular NGF levels increased significantly by more than threefold postoperatively in the OA group but not in the non-OA group. Moreover, the levels of inflammatory cytokines and lipid mediators were increased in the synovial fluid of both groups. The intra-articular cytokines or NGF concentrations positively correlated with postoperative pain. Targeted NGF control has the potential to alleviate postsurgical pain in TKA, especially in patients with OA, emphasizing the importance of understanding NGF dynamics under different knee conditions.

## Introduction

Total knee arthroplasty (TKA) is a valuable procedure that can alleviate pain; however, the development of chronic postsurgical pain remains a concern^1–3^. Strict early postoperative pain management and accelerated recovery after surgery have been reported to contribute to the avoidance of postoperative pain and the promotion of functional recovery^4,5^. Control of perioperative pain has been emphasized to reduce chronic postsurgical pain and improve the clinical outcomes of TKA.

Nerve growth factor (NGF) has attracted attention as a master regulator of chronic pain and a molecule that controls arthritis-induced pain. In particular, NGF is reported to be involved in chronic musculoskeletal inflammatory disorders such as degenerative spondylosis^6^, systemic lupus erythematosus^7,8^, rheumatoid arthritis (RA)^9,10^, and osteoarthritis (OA)^11,12^. In these diseases, NGF concentrations are elevated both in serum and synovial fluid, suggesting that NGF may locally and directly control arthritis and articular pain^13^. The expression of NGF varies depending on the disease, and it has been reported that the expression level of NGF in the synovial fluid cells and synovial tissue is higher in RA compared to OA^14,15^.

Intrusion into deep tissues such as the muscles and fascia has been reported to enhance pain via NGF signaling^16,17^. Tissue damage due to surgical invasion also leads to NGF production at the site of injury, creating hyperalgesia in rats^18^. However, no relationship has been suggested between chronic postoperative neuropathic pain and the production of TNF-α and NGF in the skin^19^. The effect of surgical invasion on NGF production has not yet been elucidated.

This study aimed to evaluate the concentration versus time profiles of NGF and related molecules in synovial fluid and plasma before and after TKA surgery and to elucidate the involvement of NGF in postoperative pain in OA and non-OA.

## Results

The demographics of all patients enrolled in this study are shown in Table 1. The mean age of the patients was approximately 70 years, with more than 80% women in both the OA (71.5 years, 81.8% women) and non-OA (67.4 years, 89% women) groups. Concomitant medication use before TKA was higher in the non-OA group than in the OA group. However, analgesic use was higher in the OA group than in the non-OA group after TKA. The pain visual analog scale (VAS) was similar between the OA (63 ± 6) and non-OA groups (71 ± 6) before TKA.

**Table 1:** Patient demographics.

Category	OA (n = 11)	Non-OA (n = 9)
	Mean (SD), unless otherwise specified	
Age (years), (range)	71.5 (63–79)	67.4 (46–77)
Sex, female, (%)	9 (81.8)	8 (89)
Diagnosis and classification	KL-3: 1 (9.1)	RA: 10 (89)
	KL-4: 10 (90.9)	SLE: 1 (11)
Preoperative medication		
Concomitant medication use, n (%)		
Prednisone	2 (18.2)	5 (55.6)
Methotrexate		6 (66.7)
Biologics^a^		3 (33.3)
Others^b^		5 (55.6)
Number of postoperative NSAIDs use, n (%)		
0	1 (9.1)	3 (33.3)
1	8 (72.7)	3 (33.3)
2	2 (18.2)	3 (33.3)
Postoperative opioids^c^ use, n (%)	2 (18.2)	0 (0)
Pain (VAS), mean ± SE		
Pre	63 ± 6	71 ± 6
Day 1	45 ± 6	54 ± 9
Day 2	27 ± 5	45 ± 10
Day 14	18 ± 4	16 ± 6

*KL* Kellgren and Lawrence^31^, *RA* rheumatoid arthritis, *SLE* systemic lupus erythematosus.

^a^Biologics include tocilizumab and certolizumab pegol.

^b^Others include bucillamine, tacrolimus, and tofacitinib.

^c^Tramadol, Tramadol with acetaminophen.

**Figure 1:**
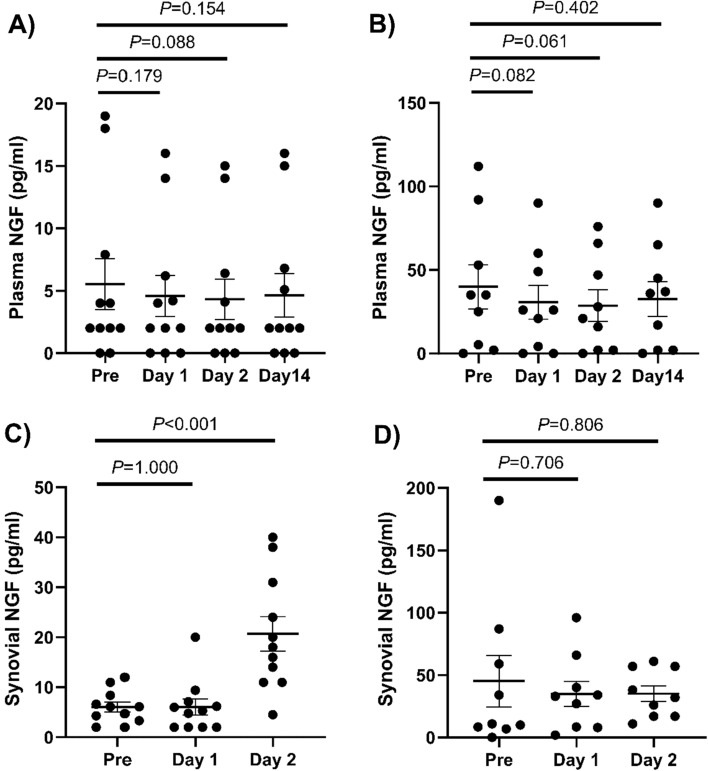
Concentration of nerve growth factor in plasma (**A**,**B**) and synovial fluid (**C**,**D**) at each time point. Plasma nerve growth factor concentrations in the OA (**A**) and non-OA groups (**B**). Nerve growth factor concentrations in synovial fluid in the OA (**C**) and non-OA groups (**D**).

**Figure 2:**
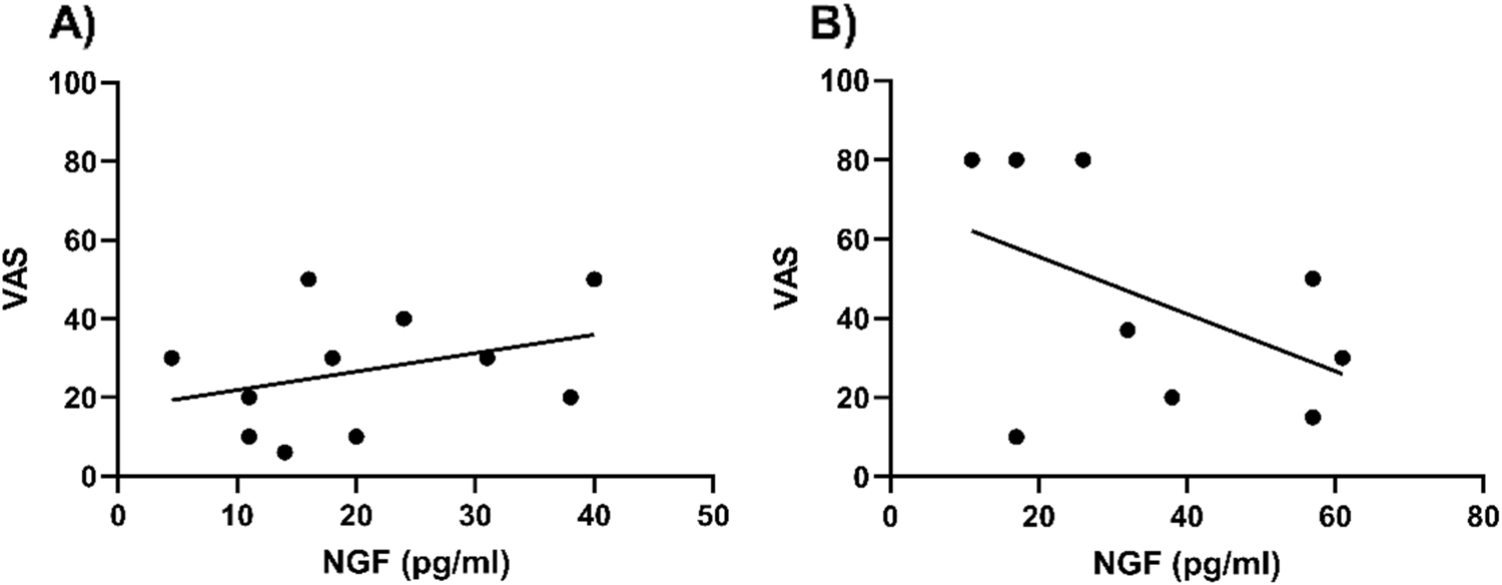
Relationship between the concentration of nerve growth factor in synovial fluid and visual analog scale at postoperative day 2 in the OA (**A**) and non-OA groups (**B**).

The plasma NGF levels in the non-OA group were more than six times higher than those in OA group during all observation periods (preoperative: OA 5.5 pg/mL vs non-OA 39.9 pg/mL, *P* = 0.011), whereas in no significant increase in NGF concentration was observed between the observation periods (Fig. 1A, B, Supplementary Table 1) in both groups. The intraoperative synovial fluid NGF concentration was more than seven times higher in the non-OA group than in the OA group (OA 6.0 pg/mL vs non-OA 45.1 pg/mL, *P* = 0.049) (Fig. 1C, D). The concentration of NGF in the synovial fluid was significantly increased postoperatively in the OA group (day 1: 6.1 pg/mL, day 2: 20.7 pg/mL, *P* < 0.001), while that in the non-OA group did not change significantly between observation periods (day 1:34.9 pg/mL, day 2: 35.1 pg/mL). The OA group showed a positive correlation between pain and NGF concentration (*r* = 0.3497), while the non-OA group showed a negative correlation (*r* =  − 0.4799) (Fig. 2).

The OA and non-OA groups showed no significant difference in plasma TNF-α and IL-6. In both groups, TNF-α concentrations gradually increased over time after surgery, while the IL-6 concentrations were highest on postoperative day 1, before decreasing thereafter (Table 2). The results for inflammatory cytokines (IL-1β, TNF-α, and IL-6), pain-inducing lipid mediators (prostaglandin E2 [PGE2]), transient receptor potential vanilloid 1 (TRPV1) endogenous agonist, 12-hydroxyeicosatetraenoic acid (HETE), and 15-HETE in synovial fluid are shown in Table 3. The concentration of TNF-α in intraoperative synovial fluid was higher in the non-OA group than in the OA group, Although there were no significant differences in other periods or other molecules between the two groups, the mean concentrations of all inflammatory cytokines and PGE2 were higher in the non-OA group than those in the OA group, whereas the mean cytokine levels in the effluent were higher in the OA group than those in the non-OA group on postoperative day 2. The 12-HETE and 15-HETE concentrations increased significantly after TKA in both groups.

**Table 2:** NGF-related cytokines in plasma and synovial fluid.

	Plasma				Synovial fluids		
	Pre	Day 1	Day 2	Day 14	Pre	Day 1	Day 2
Cytokines							
TNFα							
OA (n = 11)	1.0 ± 0.1	0.9 ± 0.1	1.2 ± 0.1	1.3 ± 0.2	0.4 ± 0.1	3.5 ± 0.6	5.6 ± 1.4
*P* (vs pre)		0.326	0.114	0.002		< 0.001	0.008
Non-OA (n = 9)	1.1 ± 0.2	1.2 ± 0.2	1.5 ± 0.3	1.6 ± 0.2	2.3 ± 0.9*	3.9 ± 1.1	3.6 ± 0.9
*P* (vs pre)		0.643	0.009	0.094		< 0.001	0.123
IL-1β							
OA (n = 11)	n.t	n.t	n.t	n.t	0.5 ± 0.5	43 ± 15	685 ± 286
*P* (vs pre)						0.032	0.067
Non-OA (n = 9)	n.t	n.t	n.t	n.t	3.6 ± 2.4	31 ± 24	144 ± 62
*P* (vs pre)						0.412	0.087
IL-6							
OA (n = 11)	4.0 ± 0.9	231 ± 44	121 ± 30	11 ± 3.9	87 ± 34	2.6 ± 0.9 (× 10^4^)	2.4 ± 0.4 (× 10^5^)
*P* (vs pre)		0.001	0.008	0.125		0.041	< 0.001
Non-OA (n = 9)	7.3 ± 2.8	137 ± 19	26 ± 5.6	11 ± 2.8	418 ± 208	2.8 ± 0.7 (× 10^4^)	1.3 ± 0.3 (× 10^5^)
*P* (vs pre)		< 0.001	0.074	0.564		0.008	0.006

*P < 0.05 (OA vs non-OA).

**Table 3:** Lipid mediators in plasma and synovial fluid.

	Plasma				Synovial fluids		
	Pre	Day 1	Day 2	Day 14	Pre	Day 1	Day 2
Lipid mediators							
PGE2							
OA (n = 11)	1.5 ± 0.8	n.d	n.d	2.3 ± 1.2	4.1 ± 2.0	29 ± 14	225 ± 71
*P* (vs pre)		0.188	0.188	0.938		0.186	0.020
Non-OA (n = 9)	0.7 ± 0.7	n.d	1.0 ± 0.9	0.3 ± 0.3	35 ± 29	34 ± 16	68 ± 30
*P* (vs pre)		0.644	0.964	0.644		0.999	0.656
12-HETE							
OA (n = 11)	0.3 ± 0.1	0.4 ± 0.1	0.2 ± 0.0	0.4 ± 0.1	0.1 ± 0.1	20 ± 3	8.0 ± 1.1
*P* (vs pre)		0.591	0.656	0.655		< 0.001	< 0.001
Non-OA (n = 9)	0.2 ± 0.1	0.4 ± 0.2	0.2 ± 0.1	0.2 ± 0.1	0.6 ± 0.6	19 ± 3	7.7 ± 2.1
*P* (vs pre)		0.395	0.935	0.995		< 0.001	0.009
15-HETE							
OA (n = 11)	0.01 ± 0.00	0.01 ± 0.00	0.01 ± 0.00	0.01 ± 0.00	0.01 ± 0.00	0.23 ± 0.04	0.19 ± 0.02
*P* (vs pre)		0.791	0.779	0.990		< 0.001	< 0.001
Non-OA (n = 9)	0.01 ± 0.00	0.01 ± 0.00	0.01 ± 0.00	0.01 ± 0.00	0.01 ± 0.01	0.22 ± 0.03	0.20 ± 0.04
*P* (vs pre)		0.634	0.029	0.933		< 0.001	< 0.001

## Discussion

We successfully investigated the plasma and intra-articular NGF concentration changes in patients with postoperative TKA pain. In knees with OA, there was no significant change in the plasma NGF concentration before and after surgery, whereas an increase in the NGF concentration in the knee joint was observed. In contrast, no significant change was observed in intra-articular NGF concentrations before and after surgery in the non-OA group.

Several studies have investigated OA and the concentration and gene expression of NGF and inflammatory cytokines in plasma and synovial fluid. Ingale et al. reported that gene expression in synovial biopsies from patients with different grades of OA showed peaks of IL-1β, IL-15, PGE2, and NGF in early OA (Kellgren–Lawrence (KL) grades I and II)^20^. Nees et al. also reported that increases in anti-inflammatory (IL-10, IL-13) and inflammatory (IL-7, IL-12, IFN-γ) cytokines and growth factors (SCGF-β, VEGF) were significantly correlated with the severity of knee pain^21^. Furthermore, Sakurai et al. reported that the NGF concentration in the synovial fluid increased in an animal OA model and that administration of NGF antibodies resulted in pain reduction^22^. We found that intra-articular NGF levels in this study were elevated after TKA for OA and positively correlated with postoperative pain. These results suggest that postoperative control of intraarticular NGF levels can achieve perioperative pain relief.

However, the intra-articular NGF concentration after TKA between the patients with RA and those with SLE were not significantly different. One possibility is that in RA and SLE, both blood and synovial fluid NGF concentrations are elevated before surgery, making it difficult to detect alterations in NGF concentrations due to surgical intervention. Another possibility is that during TKA, joint irrigation and synovectomy resulted in a decrease in intra-articular NGF concentration, offsetting any potential postoperative increase in NGF expression due to surgery. The fact that some patients with high NGF concentrations on day 0 showed a postoperative decrease in NGF concentrations may support this hypothesis. An increase in the intra-articular NGF concentration due to surgical trauma was not observed in the non-OA group, and a negative correlation was observed between the intra-articular NGF concentration and pain. Because the intra-articular NGF concentration before TKA is considerably higher than that in OA, it may influence pain in patients who do not have OA. The average NGF concentration was significantly higher than that in OA cases, suggesting that, as in OA cases, controlling intra-articular NGF postoperatively may alleviate postoperative pain.

IL-1β and TNFα stimulate the release of NGF, which, in turn, promotes the release of IL-6^23^. Our findings suggested that surgery-induced tissue damage and inflammation increase the release of IL-1β and TNFα, consequently inducing both NGF and IL-6 release. The nerve growth factor induces pain by activating the TRPV1 channel and the PGE2 and EP2 receptors. Lipid analysis revealed an increase in the postoperative levels of PGE2 and 12-HETE/15-HETE, TRPV1 endogenous agonists. This suggests that NGF activates the EP2 receptor and TRPV1 channel while increasing endogenous ligands and enhancing pain.

Regarding inflammatory cytokines in the synovial fluid, the preoperative synovial fluid concentrations of TNF-α were higher in the non-OA group than in the OA group, suggesting a greater effect of intra-articular inflammation in the non-OA group than in the OA group. In contrast, the post-operative values of TNF-α, IL-1β, and IL-6 were similar between the two groups, possibly due to an increased inflammatory response resulting from surgical invasion in both groups. The limitation in NGF production may have been caused by the removal of NGF-producing cells, such as mast cells, by synovectomy and joint irrigation in the non-OA group. In the synovial fluid of patients with rheumatoid arthritis, inflammatory hormones such as estrogen and cortisol are found to be pervasive in high concentrations, suggesting a potential contribution to the inflammatory process^24,25^. It is conceivable that the decrease in NGF concentration in the synovial fluid post-surgery in non-OA group is possibly due to the reduction of these hormones because of synovectomy and joint irrigation. Indeed, the smaller absolute values of inflammatory cytokines in the non-OA group compared with those in the OA group may indicate this. These results suggested that not only the cytokine concentration but also the number of NGF-producing cells contribute to the NGF concentration in the knee joint after surgery. Controlling NGF-producing cells may suppress NGF release and improve clinical outcomes.

Our study has several limitations that warrant mention. Firstly, we opted for the smallest sample size ethically permissible, designed to detect a post-operative increase in NGF levels. While this size was adequate to observe significant NGF elevations in OA patients following TKA, it proved insufficient for discerning variations in RA, largely due to elevated baseline NGF levels and pronounced individual differences. Additionally, the disparity in patient backgrounds between the OA and RA groups hindered our ability to make direct comparisons. Future studies with larger sample sizes are necessary to gain a more detailed understanding of the postoperative recovery process. Secondly, the study includes the potential influence of previous surgeries and medication usage on NGF expression. Arthroscopic surgery was included assuming minimal impact^26–28^, fully isolating the impact of medications, particularly steroids in RA patients, remains a challenge. Consequently, we cannot entirely dismiss the possibility that these factors may have affected our results. Finally, although no alterations were seen in plasma NGF levels in both groups, variations in measurement times might have shown alterations related to intra-articular NGF levels. Notably, an increase of NGF concentration in synovial fluid was observed on day 2 post-surgery in the OA group, suggesting that measuring plasma NGF on day 3 could have potentially shown an increase of plasma NGF levels.

Despite these limitations, our findings contribute valuable insights into the field. The levels of cytokines and mediators involved in NGF release were increased in the knee joint after surgery. Postoperative intra-articular NGF levels increased in patients with OA, whereas no such increase in intra-articular NGF levels was observed in patients with RA or SLE. These results provide useful information for examining perioperative pain control after TKA.

## Methods

### Ethical statement

This study was conducted in accordance with the principles of the Declaration of Helsinki, International Committee on Harmonization of Good Clinical Practice Guidelines, and applicable local laws and regulations. This observational study was approved by the Institutional Review Board of our institution. Informed consent was obtained from all patients before enrolment in the study. This observational study was approved by the Hokkaido University Institutional Review Board (No. 018-0229).

### Patients and methods

The study included 20 knees of 19 patients who underwent TKA for rheumatoid arthritis (RA), systemic lupus erythematosus (SLE), or knee osteoarthritis (OA). The inclusion and exclusion criteria are shown in Table 4. Based on the number of subjects used in a similar study reported in the past^29^, we calculated that a minimum of 17 cases is needed to detect a twofold difference in NGF concentration between preoperative and postoperative day 1 or 2, with a 50% difference in the coefficient of variation standard deviation, at a power of 80% and a *P* value < 0.05. The patients were categorized into OA (OA, 11 knees) and non-OA (RA, eight knees; SLE, one knee) groups.

**Table 4:** Inclusion and exclusion criteria.

Inclusion criteria	Exclusion criteria
Adult older than 20 years and younger than 80 years at the time of consent	Severe renal disfunction
Inflammatory or non-inflammatory degenerative knee joint disease requiring TKA	Immune suppression or autoimmune deficiency disorders
Willing and able to give consent	Previous surgery involving the knee joint, except for arthroscopy
	Local or systemic infections
	Hip or ankle arthritis in the affected limb
	Pregnant or planning to become pregnant during the study period
	Severe diseases (e.g., Parkinson’s disease, Alzheimer’s disease) or neuropathic disorders
	HBV, HCV, or HIV positivity
	Any other individuals deemed unsuitable by the principal investigator

*TKA* total knee arthroplasty, *HBV* hepatitis B virus, *HCV* hepatitis C virus, *HIV* human immunodeficiency virus.

Synovial and postoperative drainage fluid were collected from each patient according to the clinical study schedule shown in Table 5. During surgery, the synovial fluid was collected using an 18G needle and syringe before making a skin incision. Surgery was performed using standard techniques and a drainage tube was placed in the joint. To prevent the degradation of NGF and related molecules, a protease inhibitor cocktail (Nacalai Tesque, Kyoto, Japan) was added in advance to the drainage bottle, and the bottle was kept ice-cooled immediately after surgery until the time of drainage removal. Postoperative drainage fluid that accumulated in the drainage bottle until the time of drainage removal was collected on postoperative days 1 and 2. The drainage tube was removed after the drainage fluid was collected on the second postoperative day. Blood sampling was performed according to the study schedule and 20 mL of blood was collected at each time point. Preoperative blood sampling was conducted 1–3 days before surgery. The 3.2% sodium citrate was used as an anticoagulant. The collected synovial, postoperative drainage fluids and bloods are centrifuged at 3000 rpm, 4 ℃, for 10 min, and the supernatant is stored at − 80 ℃.

**Table 5:** Study schedule.

Item/report	Pre-op	Intra-op	Day 1	Day 2	Day 14
Informed consent	×				
Clinical outcome assessment (VAS)	×		×	×	×
Blood sampling	×		×	×	×
Synovial fluid/drainage fluid collection		×	×	×	

Routine blood tests were conducted, including hematological (e.g., hemoglobin, white blood cell count, white blood cell differential count, platelet count, and other complete blood counts) and blood chemistry tests (e.g., ALP, total bilirubin, albumin, AST, ALT, total protein, and other general biochemical parameters, as well as HBV, HCV, and HIV) (data not shown).

The NGF concentration was measured using an ELISA kit (Biosensis, BEK-2212). Cytokines were measured by an ELISA kit for interleukin (IL)-1β (R & D Systems, DLB50), tumor necrosis factor (TNF)-α (R & D Systems, HSTA00E), and IL-6 (R & D Systems, D6050). Lipid mediators were measured using liquid chromatography–tandem mass spectrometry as described previously^30^.

### Surgical procedure

All surgeries were performed via the parapatellar approach, and the prosthesis was of the PS type (ATTUNE TKA system, DePuy Synthes, USA). The tourniquet was inflated to 280 mmHg in all cases during surgery; in OA patients, all synovial membranes were retained. However, the synovium of the anterior cortex of the distal femur was dissected subperiosteal to secure the anterior flange of the femoral component; in RA and SLE patients, the synovium covering the knee joint and all layers of hemorrhage, as well as the synovium covering the anterior cortex of the distal femur and the patellar tendon, were removed. Tourniquets were not removed prior to wound closure and application of dressings.

### Data analyses

Statistical analyses were performed using GraphPad Prism 10.1. (GraphPad Software, Inc., San Diego, CA, USA). Data are expressed as the mean ± SEM. Multiple groups were compared using one-way ANOVA followed by Dunnett’s test. Non-paired groups were compared using an unpaired *t*-test.

### Supplementary Information

Supplementary Table 1.

## Data Availability

All data generated or analyzed during this study are included in this article. The datasets used and analyzed during the current study are available from the corresponding author on reasonable request.
