# Supplementary material for: The alterations in nerve growth factor concentration in plasma and synovial fluid before and after total knee arthroplasty
Source: Sci Rep. 2024 Apr 18;14:8943. doi: 10.1038/s41598-024-59685-1 (PMC11026423; doi:10.1038/s41598-024-59685-1)
Supplement: Supplementary file 1 — Supplementary Table 1. [file 41598_2024_59685_MOESM1_ESM.docx]

Supplementary Table 1. Plasma NGF levels in the OA and the non-OA groups

|  |  |  |  |  |  |  |  |  |  |
| --- | --- | --- | --- | --- | --- | --- | --- | --- | --- |
|  |  | Plasma | | | |  | Synovial fluids | | |
|  |  | Pre | Day 1 | Day 2 | Day 14 |  | Pre | Day 1 | Day 2 |
| NGF | OA (n=11) | 5.5±2.0 | 4.6±1.7 | 4.3±1.6 | 4.6±1.7 |  | 6.0±1.0 | 6.1±1.6 | 20.7±3.5 |
|  | *P* (vs Pre) |  | 0.179 | 0.088 | 0.154 |  |  | 1.000 | <0.001 |
|  | Non-OA (n=9) | 39.9±12.0 | 30.7±9.2 | 28.7±8.6 | 32.7±9.4 |  | 45.1±18.6 | 34.9±9.1 | 35.1±5.8 |
|  | *P* (vs Pre) |  | 0.082 | 0.061 | 0.402 |  |  | 0.706 | 0.806 |
|  |  |  |  |  |  |  |  |  |  |
